# Transient exposure to environmentally realistic concentrations of di-(2-ethylhexyl)-phthalate during sensitive windows of development impaired larval survival and reproduction success in Japanese medaka

**DOI:** 10.1016/j.toxrep.2020.01.009

**Published:** 2020-01-24

**Authors:** Bonny Bun Ho Yuen, Anna Boya Qiu, Bruce Hao Chen

**Affiliations:** Environmental Science Programme, Division of Science and Technology, Beijing Normal University, Hong Kong Baptist University, United International College, 2000 Jintong Road, Tangjiawan, Zhuhai, Guangdong Province, PR China

**Keywords:** DEHP, Endocrine disruptor, Embryos, Reproduction, *Oryzias latipes*

## Abstract

Di-(2-ethylhexyl) phthalate (DEHP) is a well-known endocrine disruptor and it is ubiquitously distributed in the environment. However, very few studies have investigated the effects of short-term exposure to environmentally realistic concentrations of DEHP during early developmental stages and its chronic effects. This study monitored the long-term effects of transient exposure to DEHP in early life stages (F_0_ generation) and its subsequent fertilization success in F_1_ generation using Japanese medaka, *Oryzias latipes*, as model organism. Embryos (4 h post-fertilization, 4 hpf) of Japanese medaka were exposed to 0.001 ppb, 0.1 ppb, or 10 ppb DEHP for 21 days and returned to control water (without DEHP) for maturation (4 months old). At day 9 of the exposure study, mortality was significantly increased in medaka embryos (before hatching) treated with 0.001 ppb and 10 ppb DEHP. Continual exposure of young hatchlings for an additional 12 days (a total of 21 days of exposure) resulted in a significant increase in mortality in fish exposed to 0.001 ppb, 0.1 and 10 ppb DEHP. Significant reduction in egg production was observed in adult female medaka (4 months old) with prior exposure to 0.1 ppb and 10 ppb DEHP for 21 days during early development. Fertilization and hatching success were also significantly reduced in breeding pairs with prior exposure to 0.001 ppb, 0.1 ppb and 10 ppb DEHP during early life stage. Histological analysis of adult male gonads revealed a significant decline in mature sperm count accompanied by an increase in interstitial space in fish exposed to 0.1 ppb and 10 ppb DEHP during early development. Likewise, the amount of vitellogenic (mature) oocytes observed in the ovaries of adult female with transient exposure to 0.1 ppb and 10 ppb DEHP was significantly reduced when compared with the solvent control group. Our data suggest that transient exposure to ultra low concentrations of DEHP during sensitive time windows of development results in irreversible reproductive impairment which may impact fish populations negatively.

## Highlights

• Non-monotonic dose response relationship was detected in embryos exposed to DEHP.
• Transient exposure to 0.1 ppb DEHP during early development inhibits oogenesis.
• Transient exposure to 0.1 ppb DEHP during early development inhibits spermatogenesis.
• Exposure to 0.001 ppb DEHP during early development reduces fertilization success.
• Exposure to 0.001 ppb DEHP during early development reduces hatch rate in F_1_.

## Graphical abstract

**Figure:**
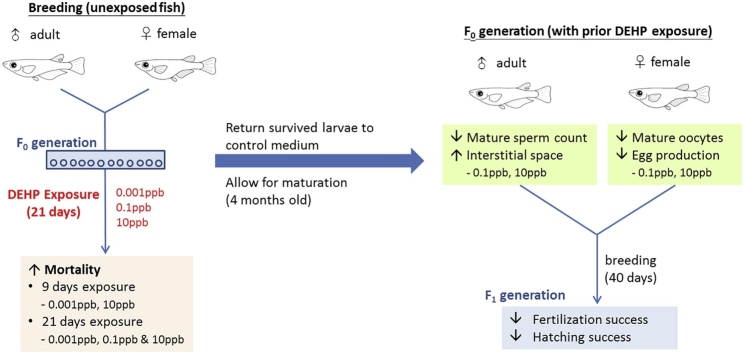

## 1 Introduction

In orderto increase the flexibility of rigid polymers, such as polyvinyl chloride, pthalate esters are commonly employed as plasticizers by the plastic industry. These chemicals are commonly found in wire and cables, self-adhesives, medical devices, cosmetics, food packages and many househould and personal care products [1]. Phthalate esters are well-known endocrine disrupting chemicals and their effects on male fertility (altered sperm count and motility), reproductive diseases in men and women (cryptorchidism and endometriosis), cardiovascular diseases, obesity, respiratory and behavioral problems have been well documented [2]. As one of the most widely used plasticizer, di-(2-ethylhexyl) phthalate (DEHP) is produced at more than 2 million tons annually [3]. Since DEHP is non-covalently bound to plastics, it leaches out very easily and pollutes the environment. Due to its estrogenic and anti-androgeniceffects, in the past two decades, many studies have investigated the effects of DEHP on the reproductive systems in mammalian species [4,5]. Although a few studies have reported inhibitory effects of DEHP on the aquatic organisms, these experiments were often conducted at extremely high concentrations, i.e., ppm levels, which cannot mimic the real environmental conditions these aquatic organisms are exposed to. For example, disturbance of spermatogenesis and peroxisome proliferation was reported in zebrafish injected with 5000 mg/kg DEHP intraperitoneally [43], and long-term exposure to 0.5 ppm DEHP has been shown to suppress immune function and spermatogenesis in Japanese medaka and catfish [[6], [7], [8]]. Since the highest dissolvable concentration of DEHP in water is reported to be around 3 ppb [9], ecotoxicological studies concerning the effects of DEHP ought to be conducted at environmentally realistic concentrations, so that policy makers can make effective measures to better protect our environment based on relevant data. Table 1 summarizes ranges of DEHP concentrations which have been detected in the water column in China and Europe in the past two decades. In China, DEHP in the water column was found to range from 0.004 ppb to 90 ppb and between 0.05 ppb–4.3 ppb in some parts of Europe (Table 1).

**Table 1:** Reported DEHP concentrations found in the surface waters in China and some countries in Europe in the past two decades.

Location	DEHP (ppb)	Reference
China		
Guangzhou (Urban lakes)	0.17	[10]
Jiulong River	0.79 – 10.9	[11]
Three Gorges Reservoir	0.004 – 1.17	[12]
Yangtze River, Wuhan Region	0.034–91.22	[13]
Yangtze River Estuary	0.61 – 28.55	[14]
Thailand	8.64	[15]
Europe		
Rieti District, Italy	4.30	[16]
Berlin, Germany	2.27	[17]
Germany	0.05 – 0.06	[18]
Greece	0.93	[18]
Dutch coast, the Netherland	0.28 – 0.65	[19] [20];

Although DEHP has been shown to impair reproduction, reduce sperm quality and lower testosterone levels in several fish species, such as zebrafish, goldfish and fathead minnow, majority of these studies only looked at the effects of DEHP on the adults [21,22]; Golsan et al., (2015). Since DEHP is a well-known endocrine disruptor, more emphasis should be placed on its effects on early development. In this study, embryos of Japanese medaka were used to assess the effects of an exposure to DEHP for the reasons that (i) embryos and larval fish have limited ability to migrate to cleaner water; (ii) many organs and physiological systems are still undergoing rapid development in these early developmental stages; and (iii) lack of fully developed phase I and phase II detoxification systems in the developing organs may lead to a reduced detoxification and elimination of toxicants [23]. Exposure to DEHP during these sensitive windows of development may result in adverse effects of high magnitudes, leading to irreversible damage and ultimately affecting the fitness of adult fish and the population. Currently, the potential long-term effects of transient exposure to environmentally realistic concentrations of DEHP to embryos and young hatchlings remain poorly investigated.

In this study, embryos of Japanese medaka, *Oryzias latipes*, were exposed to three concentrations of DEHP (0.001 ppb, 0.1 ppb and 10 ppb) for 21 days since 4 h post fertilization (4 hpf). The objectives of the present study were to investigate (i) if exposure to environmentally realistic concentrations of DEHP affect survival of embryos and young hatchlings, and the time fertilized eggs requires to hatch (hatching time), (ii) if transient exposure to environmentally realistic concentrations of DEHP during early developmental stages permanently affects reproduction success in adult fish in terms of egg production, fertilization and hatching success; and (iii) if transient exposure to environmentally realistic concentrations of DEHP during early life stage permanently alters oogenesis and spermatogenesis. Japanese medaka was chosen as the model-organism due to (i) the transparency of the medaka’s eggs allows embryonic development to be observed without sacrificing the embyros; (ii) extensive information is available of the physiology, embryology and genetics of Japanese medaka, and (iii), the ease of culture of this species in the laboratory, such as daily spawning, external fertilization and relatively short generation time (approximately 3 months) [24,25].

## 2 Methods

All animal procedures were conducted in accordance and approved by the UIC Animal Care and Use Committee.

### 2.1 Chemicals

DEHP was obtained from Dr. Ehrenstorfer GmbH (Germany, analytical grade). Stock solutions of DEHP (5 ppb, 500 ppb and 50 ppm) were prepared by dissolving DEHP in acetone (Sigma Aldrich, Germany, analytical grade), and the final acetone concentration in the exposure water was 0.02 % (v/v), i.e., 200 μl of each stock solution (5 ppb, 500 ppb and 50 ppm) was added into 1 L embryo rearing medium (ERM) to prepare 0.001 ppb, 01 ppb and 10 ppb DEHP working grade solutions. Solvent control contained acetone only.

### 2.2 Animals and exposure studies

Adult Japanese medaka, *Oryzias latipes*, were cultured in flow-through tanks under a constant 14 h/10 h light/dark cycle at 24 ± 2 °C, with pH maintained at 7.4 ± 2. Adult fish were fed twice daily with Otohime Fish Diet (PTAqua, Japan) and fresh *Artemia nauplii*. During breeding, adult medaka, i.e., breeding pairs (1 male and 1 female), were raised in 1X Embryo Rearing Medium (ERM) prepared by diluting 25 mg of neomycin sulfate, 50 μl stock solution (35 % w/v) of sodium thiosulfate, 125 μl Amquel® and 200 μl of stock solution (0.1 % w/v) of methylene blue in 1 L of pure water.

#### 2.2.1 Range-finding test

Eggs from 10 breeding pairs were pooled and sorted using stereomicroscope (Leica, USA). Transparent eggs with doubled membrane (approximately 4 hpf) were assigned to petri dishes each containing 30 fertilized eggs for exposure study. Preliminary range finding test on DEHP on embryonic toxicity were conducted by exposing 4 hpf embryos to 0.0001 ppb, 0.001 ppb, 0.01 ppb, 0.1 ppb, 1 ppb and 10 ppb DEHP, with control and solvent control groups for 9 days (i.e., prior hatching). Nominal concentration was used in all cases. Solutions were renewed daily throughout the 9-day exposure period and a total of 90 fertilized eggs (30 eggs/petri dish, 3 petri dishes per group, n = 3) were used for each control and treatment groups. Based on the results obtained from the range finding test, subsequent long-term monitoring (5 months) on the effects of transient exposure to DEHP during early developmental stages were conducted in survived hatchlings with prior exposure to 0.001 ppb, 0.1 ppb and 10 ppb DEHP.

#### 2.2.2 Long-term study

Fertilized eggs (4 hpf) were exposed to control group, solvent control group (acetone at a final concentration of 0.02 % [v/v]), 0.001 ppb DEHP, 0.1 ppb DEHP and 10 ppb DEHP treatment groups for 21 days. Nominal concentration was used in all cases. Solutions were renewed daily throughout the 21-day exposure period and a total of 90 fertilized eggs (30 eggs/petri dish, 3 petri dishes per group, n = 3) were used for each control and treatment groups. After 21-day exposure, surviving larvae/young hatchlings were returned to ERM (minus DEHP and solvent) and flow-through tanks for maturation (to reach 4 months of age). Temperature, light/dark cycle, pH and feeding regimes were identical as those used for brood stocks.

### 2.3 Reproduction study

At 4 months old, adult medaka which have been transiently exposed to DEHP during early development were arranged in breeding pairs for the reproduction study. To breed, one male and one female adult medaka were placed in 1 L of 1X ERM and were allowed to breed freely for 40 days. Five pairs of adult fish (n = 5) were used for each control and treatment groups with ERM renewed daily. Eggs were collected twice daily 1 h after the mating pairs were fed with fresh *Artemia nauplii*. Stereomicroscope was used to record the number of eggs produced and fertilization success (i.e., the presence of double membrane as perivitelline space could be observed after successful fertilization) per breeding pair. Fertilized eggs were placed in petri dishes containing 1X ERM and observed for hatching success (9-day observation period). ERM was renewed daily and dead embryos were discarded.

### 2.4 Morphological analysis

At the end of the experiment, five male (n = 5) and five female (n = 5) adult medaka (approximately 5 months old) for each control and treatment groups were dissected. Their gonads were fixed in 4 % paraformaldehyde, dehydrated in graded ethanol series, cleared in xylene and embedded in paraffin. Tissues were sectioned at 5 μm thickness and stained by Hematoxylin and Eosin for morphological analysis using a Leica light microscope (Leica, USA). Images of male and female gonads were captured by SPOT Insight Color Digital Camera with SPOT Software (SPOT, USA). Image Pro Plus (IPP) 6.0 (Media Cybernetics, USA) was used to manually point count different cell types. Five images (20X magnification for ovaries and 40X magnification for testes) were captured randomly per fish and used for quantitative analyses. A digital grid mask (24*18 = 432) intersection points from IPP was superimposed on each image. For testes, spermatocyte/spermatogonia, spermatids/spermatozoa and interstitial space were counted in red, blue and green dots, respectively. For ovaries, stage I oocytes (perinucleolar oocyte), stage II oocytes (cortical alveolus stage) and stage III oocytes (vitellogenic oocytes) were counted in red, green and blue dots, respectively.

Percentage of each cell type was calculated using the formula

% cell type = dots superimposed on the cell type /all dots superimposed on testis or ovary per image

### 2.5 Statistical analysis

One-way analysis of variance was used to test the null hypotheses that exposure to different waterborne concentrations of DEHP did not cause significant changes in (i) mortality and hatching success of embryos and hatchlings during the 9-day and 21-day exposure periods; (ii) spermatogenesis in adult male medaka transiently exposed to DEHP during early development; and (iii) oogenesis in adult female medaka transiently exposed to DEHP during early development. Two-way analysis of variance was used to test the null hypothesis that transient exposure to different concentrations of DEHP during early developmental stages do not cause significant changes in the amount of (i) eggs produced, (ii) eggs fertilized and (iii) eggs hatched by these adult fish. If a significant difference (*p* ≤  0.05) between the controls and DEHP treatment groups was identified, pairwise comparisons between each individual treatment and control groups were carried out using the Holm-Sidak method. Likewise, whenever significant difference (*p* ≤  0.05) was detected within the same control and/or treatment group, pairwise comparisons on the amount of eggs produced, amount of eggs fertilized and successful hatch outs were carried out using the Holm-Sidak method. In cases where data failed to follow Gaussian distribution, log_10_ or arcsin squart root transformation would be performed prior to analysis. Statistics were performed using the statistical software SigmaPlot, Version 12 (Systat, USA). Graphs were plotted using Graphpad Prism, Version 7.00 (Graphpad Software, Inc.).

## 3 Results

### 3.1 Mortality of 9 days old embryos

**Fig. 1:**
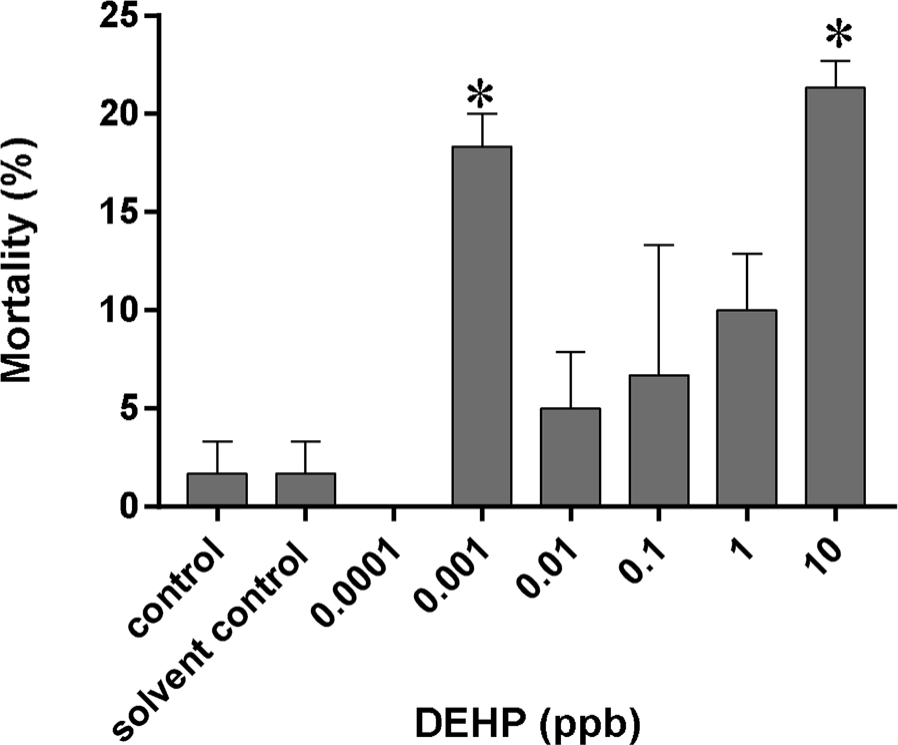
Mortality of medaka embryos after exposure to 0.0001 ppb–10 ppb DEHP for 9 days since 4 hpf. Data are expressed as mean ± standard error (n = 3). * *p* ≤  0.05 *vs*. control and solvent control.

Background mortality was found at 1.7 ± 1.7 % for both the control and solvent control groups at day 9 of the exposure study (Fig. 1). Day 9 was chosen in this experiment for the detection on embryonic toxicity as it is close to the hatching stage of Japanese medaka, before the fry escaped from the protection of the chorion [26]. Mortality was significantly increased in embryos exposed to 0.001 ppb (18.3 ± 1.7 %) and 10 ppb DEHP (21.3 ± 1.3 %) when compared with the controls (*p* ≤  0.05) (Fig. 1). No significant difference in mortality was observed between the controls and other DEHP treatment groups after 9 days of exposure (Fig. 1). Due to these preliminary findings, subsequent experiments were conducted on 0.001 ppb, 0.1 ppb and 10 ppb DEHP treatment groups only.

**Fig. 2:**
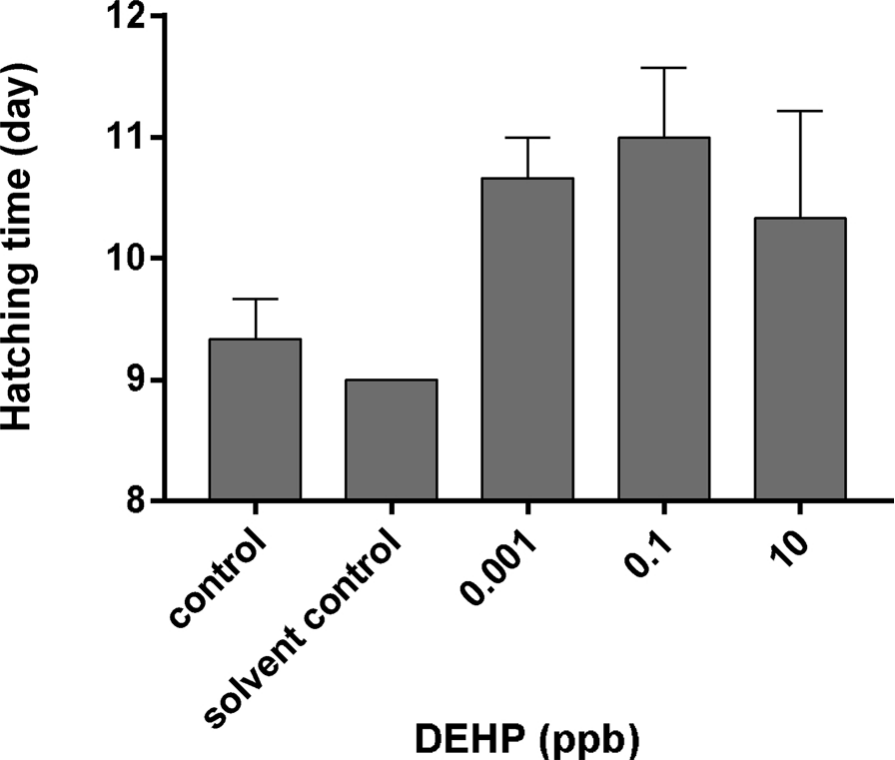
Average hatching time of medaka embryos exposed to 0.001 ppb, 0.1 ppb or 10 ppb DEHP. Data are expressed as mean ± standard error (n = 3).

Although hatching delay was observed in embryos treated with 0.001 ppb, 0.1 ppb and 10 ppb DEHP, no significant difference was detected between the treatments and the control groups (*p > 0.5*) (Fig. 2).

### 3.2 Mortality of 21 days old hatchlings

**Fig. 3:**
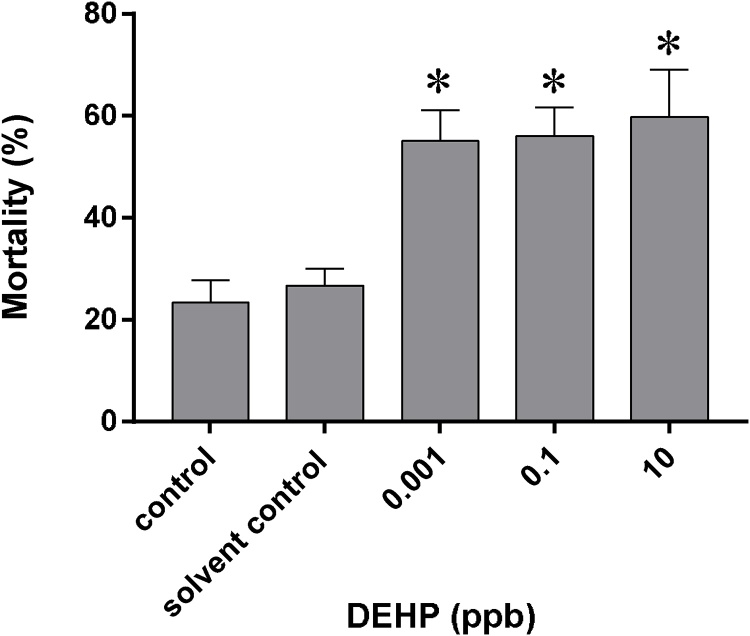
Mortality of 21-day old medaka hatchlings upon exposure to 0.001 ppb, 0.1 ppb and 10 ppb DEHP since 4 hpf. Data are expressed as mean ± standard error (n = 3). * *p* ≤  0.05 *vs*. control and solvent control.

Significant increase in mortality was observed in hatchlings exposed to 0.001 ppb, 0.1 ppb and 10 ppb DEHP for 21 days since 4 hpf, with mortalities recorded at 55.0 ± 6.0 %, 56.0 ± 5.6 % and 59.7 ± 9.4 %, respectively, when compared with the solvent control (26.7 ± 3.3 %) (Fig. 3).

### 3.3 Reproduction study on adult fish with prior exposure to DEHP during early development

**Fig. 4:**
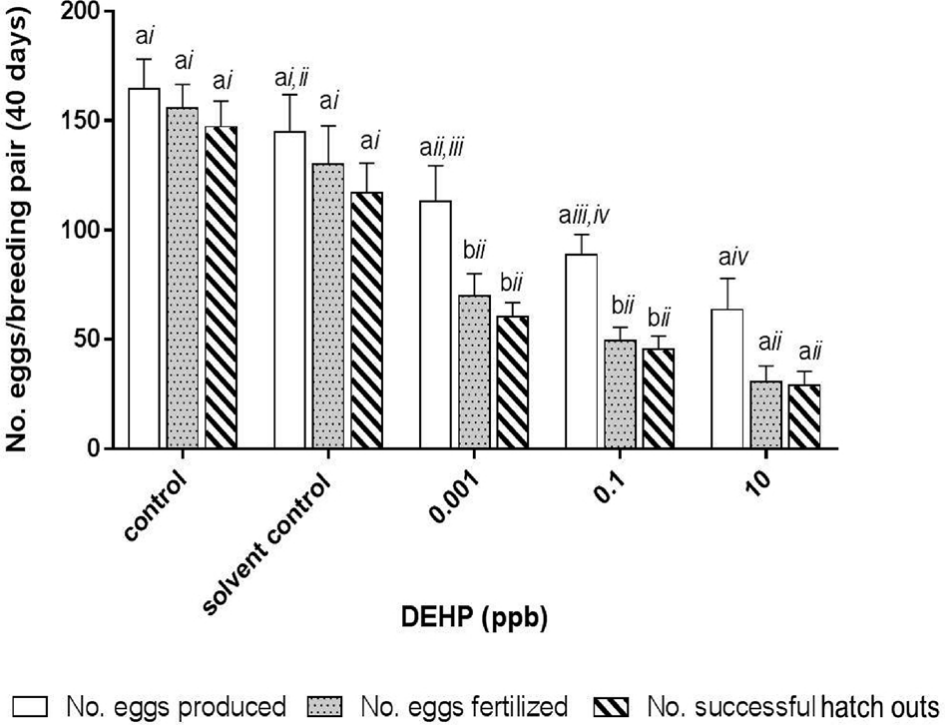
Reproduction success of adult fish with prior exposure to 0.001 ppb, 0.1 ppb or 10 ppb DEHP during early developmental stages. Total number of eggs produced, total number of eggs fertilized and total number of successful hatch outs per breeding pair were analyzed over a 40-day study period. Data are expressed as mean ± standard error (n = 5). Differences detected between no. eggs produced, no. eggs fertilized and no. successful hatch outs within the same control/treatment group marked with the same English letter (i.e., a, b) are not significantly different between one another (*p > 0.05*). The same endpoint measurement (i.e., no. eggs produced, no. eggs fertilized or no. of successful hatch outs by breeding pair with/without prior exposure to different concentrations of DEHP) marked with the same roman number (i.e., *i, ii, iii* and *iv*) indicated no significant difference was detected between the controls and other DEHP treatment groups (*p > 0.05*).

After exposure to 0.001 ppb, 0.1 ppb or 10 ppb DEHP for 21 days, survived hatchlings were returned to 1X ERM (minus DEHP and acetone) for maturation. At approximately 4 months old, a 40-day breeding study was performed on the survived adult medaka to determine if prior DEHP exposure affected reproduction success of the adult fish. Fig. 4 shows the number of eggs produced per female medaka per breeding pair (i.e., 1 male and 1 female) in a 40-day study period. Each female medaka produced, on average, 164 ± 14 and 141 ± 19 eggs during the 40-day study period in the control and solvent control group, respectively. On average, 113 ± 16, 89 ± 9, and 64 ± 14 eggs were produced per female medaka which has been transiently exposed to 0.001 ppb, 0.1 ppb or 10 ppb DEHP, respectively, during early development (i.e., from 4 hpf to 21 days old hatchling) (Fig. 4). Significant reductions in egg production were detected in female fish transiently exposed to 0.1 ppb and 10 ppb DEHP during early development, these reductions accounted for a 37 % (0.1 ppb DEHP) and 55 % (10 ppb DEHP) decrease from the total amount of eggs produced by each female medaka in the solvent control group during the 40-day study period. The reduction in egg productions appeared to be dose-dependent.

**Fig. 5:**
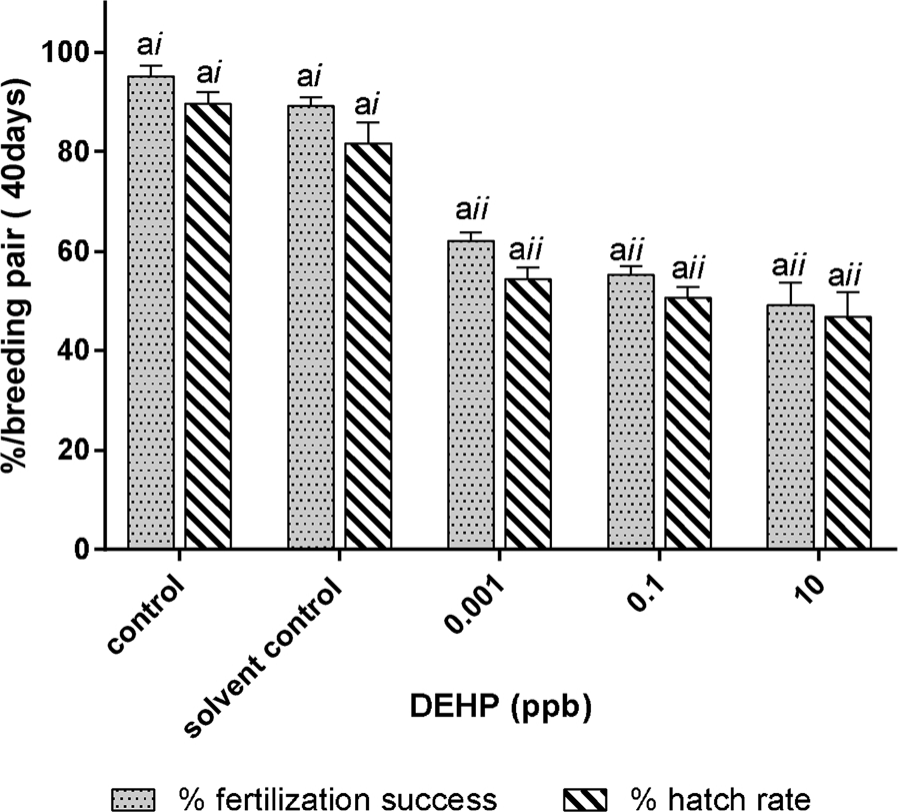
Fertilization and hatching success in adult fish transiently exposed to 0.001 ppb, 0.1 ppb or 10 ppb DEHP during early development. Percentage of fertilization success and hatch out success per breeding pair were analyzed over a 40-day study period. Data are expressed as mean ± standard error (n = 5). Differences detected between % fertilization success and hatch rate within the same control/treatment group marked with the same English letter (i.e., a) are not significantly different between one another (*p > 0.05*). The same endpoint measurement (i.e., % fertilization success, or % hatch rate) marked with the same roman number (i.e., *i or ii*) indicated no significant difference was detected between the controls and other treatment groups (*p>0.05*).

Number of eggs successfully fertilized by breeding pairs with prior exposure to 0.001 ppb. 0.1 ppb and 10 ppb DEHP during early development were significantly lower than those of the control and solvent control groups (Fig. 4). Moreover, significant declines were detected between the number of eggs produced and eggs fertilized in breeding pairs with prior exposure to 0.001 ppb, 0.1 ppb and 10 ppb DEHP during early development while no significant difference was detected among the control groups (Fig. 4). Percentage of fertilization success, i.e., no. eggs fertilized/no. eggs produced*100, was observed at 95 ± 2 % and 90 ± 2 % for the control and solvent group over a 40-day study period, respectively (Fig. 5). Transient exposure to 0.001 ppb, 0.1 ppb and 10 ppb DEHP during early development significantly reduced fertilization success to 62 ± 2 %, 55 %±2 % and 49 ± 5 %, respectively, when compared with an over 90 % success rate in both control groups (Fig. 5).

Hatching success, in terms of number of successful hatch outs, was significantly reduced in breeding pairs with prior exposure to 0.001 ppb, 0.1 ppb and 10 ppb DEHP when compared with those of the control and solvent control groups (Fig. 4). Hatch rates (i.e., no. of successful hatchings/no. of eggs produced*100) by each breeding pair during the 40-day study period were found at 90 ± 5 % and 82 ± 10 % for the control and the solvent control group, respectively (Fig. 5). Transient exposure to 0.001 ppb, 0.1 ppb and 10 ppb DEHP during early development significantly reduced hatch rates to 54 ± 5 %, 51 ± 5 % and 47 ± 11 %, respectively, when compared with the control and solvent control groups (Fig. 5).

Although within each treatment group (0.001 ppb, 0.1 ppb and 10 ppb DEHP), the number of successful hatch outs was significantly lower than the number of eggs produced by the breeding pair (Fig. 4), no significant difference was detected between the number of eggs fertilized (% fertilization success) and number of successful hatch outs (% hatch out) within each treatment group (Fig. 4, Fig. 5).

### 3.4 Effects of DEHP on oogenesis

**Fig. 6:**
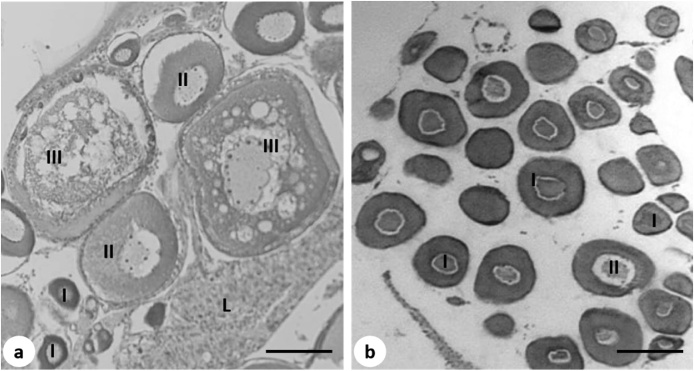
Light micrographs (H&E stain) of the gonads of 5-month old female medaka transiently exposed to DEHP during early development. (a) Solvent control and (b) 0.1 ppb DEHP treatment group. I, stage I oocytes (perinucleolar oocytes); II, stage II oocytes (cortical alveolar oocytes); III, stage III oocytes (vitellogenic oocytes); L, liver. Scale bar = 20 μm.

**Fig. 7:**
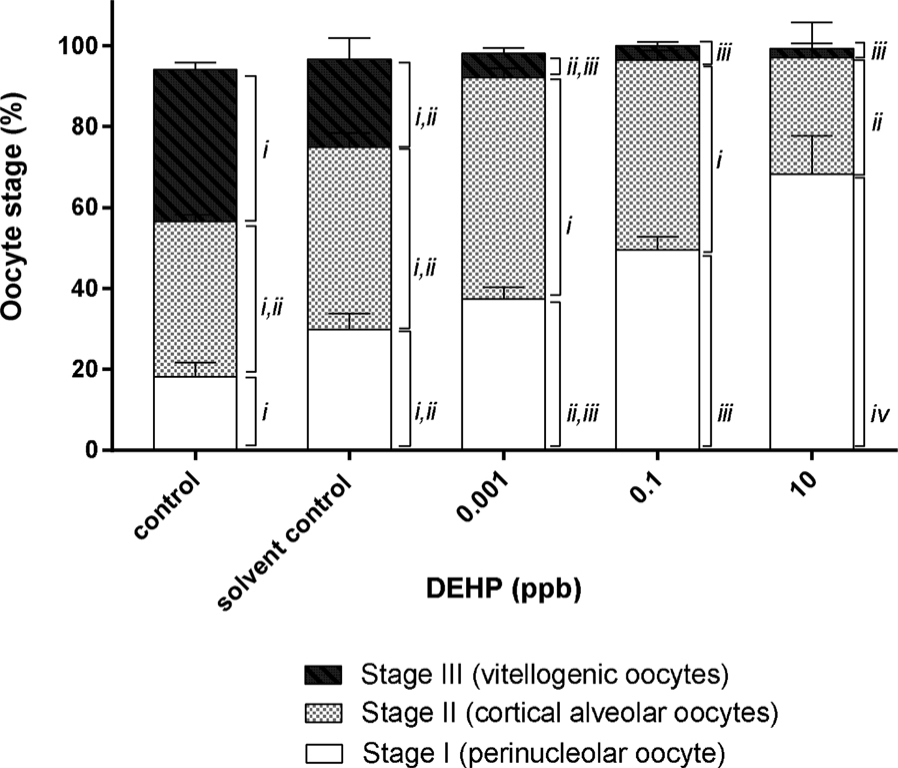
Ovary maturation stages in gonads of female medaka transiently exposed to 0.001 ppb, 0.1 ppb and 10 ppb DEHP during early development. Data are expressed as mean ± standard error (n = 5). Percentage of eggs in the same maturation stage (i.e., stage I, II or III) between different control and treatment groups marked with the same roman number (*i, ii, iii* or *iv*) are not significantly different from one another (*p > 0.05*).

Histological analysis revealed that pre-exposure to 0.1 ppb and 10 ppb DEHP during early development significantly affected oogenesis in female medaka (Fig. 6b and 7 ). Transient exposure to DEHP significantly increased the percentage of stage I oocytes (perinucleolar oocytes) in female gonads from 29.9 ± 4.0 % in the solvent control group to 49.5 ± 3.2 % in the 0.1 ppb DEHP and 68.2 ± 9.5 % in the 10 ppb DEHP treatment group (Fig. 7). Although the percentage of stage II oocytes (cortical alveolar oocytes) was not significantly different between the solvent control and the treatment groups, percentage of stage III oocytes (vitellogenic oocytes) was significantly reduced from 21.7 ± 5.3 % in the solvent control to 3.6 ± 0.8 % in the 0.1 ppb DEHP and 2.2 ± 1.3 % in the 10 ppb DEHP treatment groups (Fig. 7).

### 3.5 Effects of DEHP on spermatogenesis

**Fig. 8:**
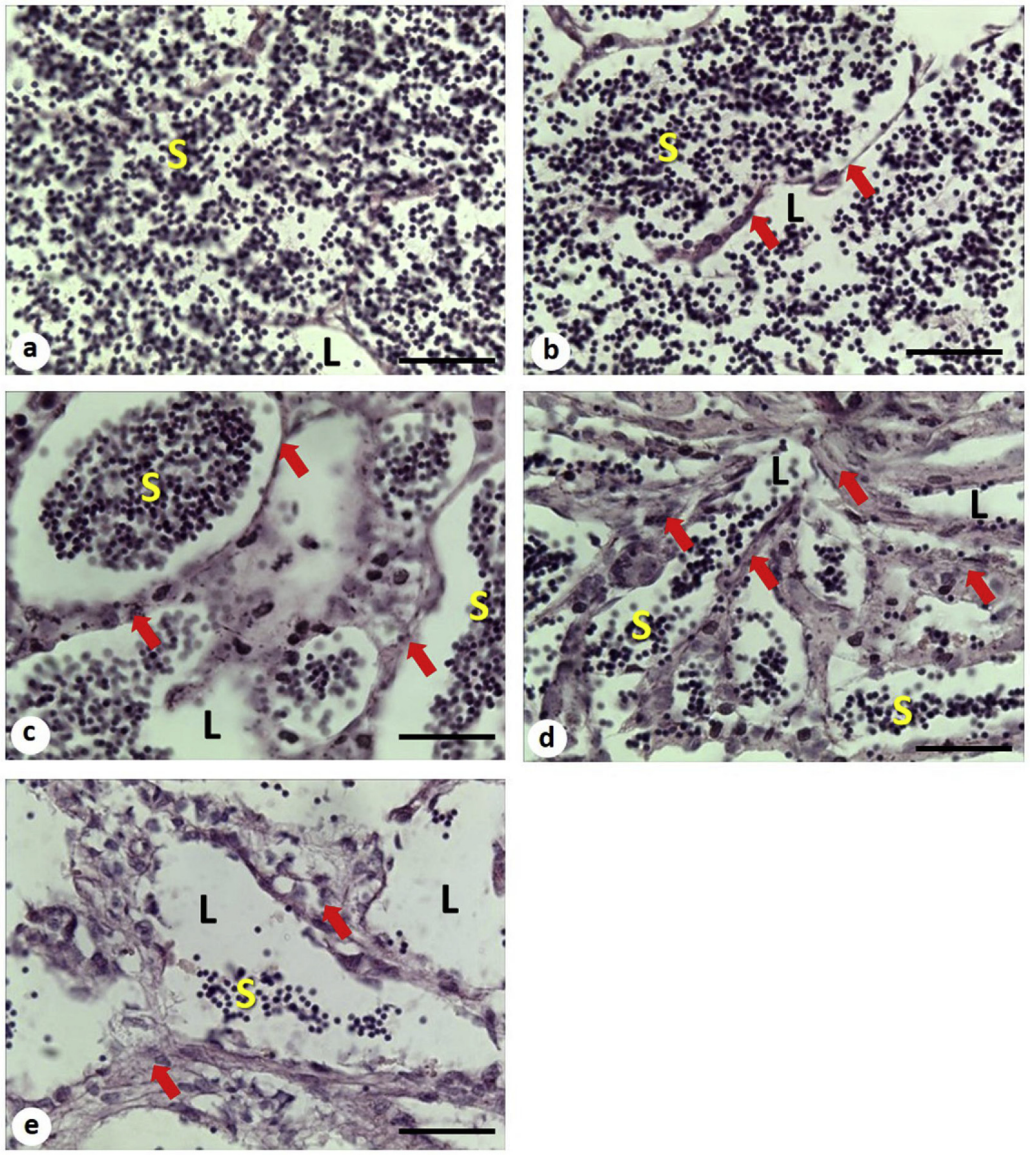
Light micrographs (H&E stain) of the gonads of 5-month old male medaka transiently exposed to 0.001 ppb, 0.1 ppb and 10 ppb DEHP during early development. (a) control, (b) solvent control, (c) 0.001 ppb DEHP, (d) 0.1 ppb DEHP and (e) 10 ppb DEHP. Arrows indicate interstitial tissues; L, lumen of tubule; S, spermatids / spermatoza. Scale bar = 5 μm.

**Fig. 9:**
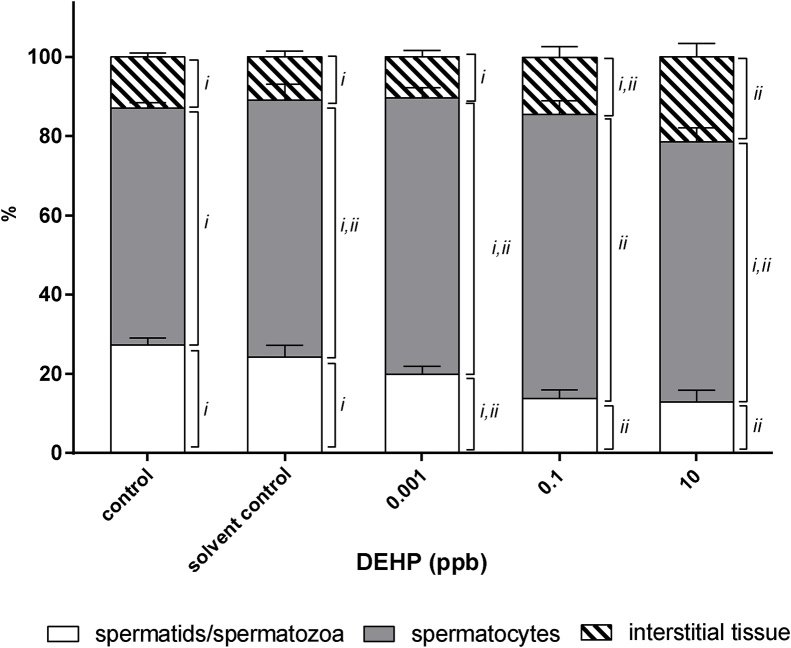
Different stages of spermatogenesis in testes of male medaka transiently exposed to 0.001 ppb, 0.1 ppb and 10 ppb DEHP during early development. Data are expressed as mean ± standard error (n = 5). The same cell type between different treatment groups marked with the same roman number are not significantly different between one another (*p > 0.05*).

A histo-morphological inspection of testes revealed that transient exposure to DEHP during early development significantly reduced mature sperm (spermatozoa and spermatids) count from 27 ± 4 % in the control and 24 ± 6 % in the solvent control to 14 ± 4 % and 13 ± 6 % in the 0.1 ppb and 10 ppb DEHP treatment group, respectively (Figs. 8a–e and 9 ). The lumen (L) of the seminiferous tubules was filled with mature sperm and the tubules were bounded by a thin basement membrane in testes of male fish collected from the control and solvent control groups (Fig. 8a and b), while an increase in lumen (L) void of mature sperm was observed in fish exposed to 0.001 ppb, 0.1 ppb and 1oppb DEHP (Fig. 8c–e). Although no significant difference was observed in the percentage of spermatocytes between the solvent control and DEHP treatment groups, significant thickening of interstitial tissue was observed in male medaka previously exposed to 10 ppb DEHP (21 ± 3 %) during early development when compared with the control (13 ± %) and solvent control group (11 ± 1 %) (Figs. 8e and 9).

## 4 Discussion

In the past two decades, majority of studies focused on the acute effects of DEHP in murine species [3,9,27];. Unlike mammals, aquatic organisms are exposed to waterborne pollutants continuously, making them more susceptible to DEHP toxicity than terrestrial animals. Due to its low water solubility, aquatic organisms are more likely to be exposed to extremely low concentrations of DEHP (in ppb range, rather than in ppm range). Compared with the adult fish, embryos and young hatchlings are more sensitive to endocrine disruptors because of their limited motility, lower body lipid contents and still rapidly developing organ systems. However, little information is available on the long-term effects of DEHP upon its short-term exposure during early developmental stages. Since DEHP is a well-known endocrine disruptor, the adverse effects of DEHP on larval toxicity and fish reproduction was investigated in this study by exposing embryos to environmentally realistic concentrations of DEHP for 21 days with subsequent long-term monitoring of its effects in adult fish.

### 4.1 Effects of ultralow doses of DEHP on embryonic toxicity

In contrast to most toxicological studies which exhibit monotonic dose-response relationship (i.e, linear relationship), non-monotonic dose response was observed in 9-day old medaka embryos exposed to 0–10 ppb DEHP. At the high end of the exposure spectrum, i.e., 10 ppb DEHP, mortality was significantly higher than that of the control groups. As the dose diminished, i.e., 0.01 ppb–1 ppb DEHP, mortality of 9-day old medaka embryos decreased to that of the control level. Further reduction in DEHP concentration, i.e., at 0.001 ppb, however, resulted in significantly higher mortality than those of the control groups (Fig. 1). Non-monotonic dose-response relationships have been reported in a few occasions, particularly with studies investigating the effects on radiation and endocrine disruptors [28]. Hooker et al., [29] reported x-ray induced mutations in the spleen of pKZ1 mice followed triphasic dose response relationship. Likewise, higher rates of apoptosis were found in zebrafish embryos irradiated with ultra-low doses of microbeam protons or x-ray when compared with those irradiated with low doses, with the rates of apoptosis climbed again at high doses of irradiation [30,31]. It is important to note that Andrade et al. [32] reported similar triphasic (or J-shaped) dose-response relationship when investigating the effects of DEHP exposure on aromatase activity in Waster rats. Aromatase activity plays a critical role in brain sexual differentiation. Significant inhibition was only detected in Waster rats when postnatal day 1 males were exposed to ultralow doses (0.135 and 0.405mgDEHP/kg/day) or high doses (15, 45 and 405mgDEHP/kg/day) of DEHP, with no significant difference detected in the low to medium doses. Our data also supports a triphasic dose response relationship between DEHP concentrations and the mortality of 9-day old medaka embryos. Although many data could explain the toxic response at high-dose exposure, little is known on the mechanisms of toxicity at ultra-low dose exposure. It has been proposed that damage at ultra-low doses may result in molecular alteration that goes undetected and hence, unrepaired (i.e., a threshold has to be triggered to initiate repair mechanisms) [33]. It is plausible that when 4 hpf medaka embryos were exposed to ultra-low dose of DEHP (0.001 ppb), molecular damages started to occur but went undetected. As cells continued to divide during embryogenesis, such damages accumulated and eventually halted the development of the embryo and resulted in death. Our findings raise the issue on the adequacy of routine hazard assessment protocols to detect these ultra-low dose effects. More importantly, as current regulation on chemicals are based on the assumption that all chemicals exhibit monotonic dose-response relationship, the lack of linear relationship between DEHP at ultra-low concentrations on embryotoxicity makes it difficult to predict the health effects at low doses using the result from high-dose exposure studies. The triphasic dose-response relationship is an intriguing area of investigation which has significant implications in environmental and health risk assessment.

Significantly higher mortality was observed in medaka hatchings exposed to 0.001 ppb, 0.1 ppb and 10 ppb DEHP 21 days since 4 hpf (Fig. 3), which contradicted to those reported by Chikae et al. [34] and Yang et al. [35], in which no significant increase in mortality was observed in medaka hatchlings/frys exposed to 0.01 ppb–200 ppb DEHP for 21 days. One major difference which could account for such discrepancy could be that both Chikae et al. [34] and Yang et al. [35] exposed hatched frys to DEHP, while in this study, embryos were exposed to DEHP since 4 hpf. The high sensitivity of medaka embryos (stage 1–39, before hatching stage) to DEHP could be the result of rapid development of organs. Since DEHP is an endocrine disruptor, its presence in the embryos would lead to hormonal imbalance, upsetting cell proliferation, apoptosis and differentiation, and eventually death of the embryos. Reduced sensitivity of young hatchlings/frys to DEHP could be the result of a more developed detoxification system. Nonetheless, current results confirmed that DEHP readily crosses the chorion and exerts its negative effects on the embryos.

Although a few studies have reported significant delays in hatch out time in medaka embryos exposed to 0.1–1 ppb DEHP [36,35], no significant change in hatch out time was detected between embryos exposed to different DEHP treatment groups and the control groups in this study (Fig. 2). Since the statistical power (0.386) of the performed test (one way ANOVA) was below 0.8 in this study, insignificant findings should therefore be carefully interpreted as to prevent Type II error. DEHP has been reported to reduce secretion of hatching enzymes in zebrafish embryos via upsetting the hypothalamus-pituitary-axis (HPT axis) [37]. It is possible that exposure to ultralow levels of DEHP could also have affected the HPT axis in embryos of Japanese medaka, thereby reducing the release of hatching enzymes leading to delayed hatching.

### 4.2 Effects of early exposure to DEHP on adult fish reproduction

Throughout the study period (approximately 5 months in length), no significant difference was found between the control and solvent control group in terms of mortality, reproduction success (i.e., eggs produced by each breeding pair, fertilization success and hatch rates) and morphological analyses of the gonads of male and female medaka, indicating that 0.02 % v/v acetone has neglectable effect on Japanese medaka. Transient exposure to 0.001 ppb, 0.1 ppb and 10 ppb DEHP during early development significantly reduced egg production in 4 month-old female medaka (Fig. 4). Similar findings have been reported in several fish species, including zebrafish, guppy, marine medaka and Chinese rare minnow, upon exposure to bisphenol A, DEHP, MEHP and 17 α-ethinylestradial (EE2) as adults [6,21,[38], [39], [40], [41], [42]]. Reduction in ovulation and oocyte maturation has been reported in adult female zebrafish exposed to 20 ppb and 40 ppb DEHP for 3 weeks via an up-regulation of bone morphogenetic protein 15 (BMP15) and a down-regulation of cyclooxygenase (COX)-2 [21]. It is possible that transient exposure to ultralow levels of DEHP during early development may have resulted in irreversible up-regulation of BMP15 and down regulation of COX-2 in female Japanese medaka, which prevented maturation and ovulation of oocytes, thus a decline in egg production. This hypothesis is in alignment with our histo-morpholoical findings, i.e., the percentage of stage I immature oocytes found in the ovaries has significantly increased from 30 % in the solvent control group to 69 % in the 10 ppb DEHP treatment group, whereas the formation of stage III vitellogenic oocytes (i.e., mature oocytes) has decreased significantly from 22 % in the solvent control group to 2 % in the 10 ppb DEHP treatment group (Fig. 7).

Transient exposure to 0.001 ppb, 0.1 ppb and 10 ppb DEHP during early development not only resulted in permanent reduction in egg production in adult female fish, fertilization success of eggs produced by these breeding pairs (both males and females with prior exposure to DEHP) was also significantly reduced (Fig. 4). The reduction could be explained by either a decline in the quality and/or quantity of eggs and sperms. A decrease in sperm quality and quantity upon exposure to DEHP has been documented in several fish species, such as Chinese rare minnow, goldfish, rainbow trout, zebrafish, as well as the marine medaka [6,[42], [43], [44], [45], [46], [47]]. Histo-morphological analysis of this study reveals a significant reduction in the quantity of mature sperms (i.e., spermatids/spermatozoa) in the gonads of male medaka with prior exposure to DEHP during early development, suggesting that less sperms were available for fertilization, leading to a decline in fertilization success (Fig. 8 & 9). DEHP has been shown to alter the expressions of several key proteins which are responsible for spermatogenesis, for example, exposure of DEHP to adult fish has been reported to alter the expression of 11 ketotestosterone [46], *boule* gene [47], peroxisome proliferator-activated receptor (PPAR) responsive genes [43], *cyp19a* in testes, and plasma 17β-estradiol and testosterone levels [6,42,48]. Exposure to ultralow doses of DEHP during early development may have triggered similar molecular changes in the gonads of male medaka, leading to a reduced sperm production. Our study clearly shows that short-term exposure to ultralow levels of DEHP during early development irreversibly damaged spermatogenesis of adult male fish.

Although exposure to DEHP has been shown to increase reactive oxygen species (ROS) production by reducing sperm motility and inhibiting follicle growth in mice and rats [[49], [50], [51], [52]], such proposed mechanism of toxicity may not be applicable in this study. Adult fish (approx. 4–5 months old) used for the reproduction study were only transiently exposed to DEHP during the first three weeks of development (i.e., from 4 hpf to 21 days old), and were raised in the absence of DEHP for 4 months before further analyses on reproduction success and histo-morphology were performed. Thus, no DEHP should be presence in the fish’s system to cause any direct oxidative damages. However, it could be possible that transient exposure to ultralow levels of DEHP during early development could permanently alter the expression of some pro-apoptotic and anti-apoptotic factors which play important roles in oogenesis and spermatogenesis. It is worthwhile to study the rate of apoptosis and expression of these pro- and anti-apoptotic genes in the gonads of adult fish with prior exposure to DEHP in future study.

Although the reduction in the number of eggs laid by female fish and fertilization success could be explained by the adverse effects of DEHP on the actions of sex steroid hormones resulting in the inhibition of oogenesis (Fig. 7) and spermatogenesis (Fig. 9), such phenomena could also be the result of altered sexual behaviors. Many studies have reported suppressed courtship activities, i.e., dancing, following, floating and crossing, in fish exposed to endocrine disrupting chemicals. For examples, altered sexual behaviors have been reported in adult fish, such as zebrafish and medaka, exposed to high concentrations of endocrine disruptors and/or 17α-ethinylestradiol ([[53], [54], [55], [56]]). It is worth investigating if transient exposure to ultra-low doses of DEHP during early developmental stages could irreversibly affect sexual behaviors in the adults. Since olfaction (detection of pheromone) and vision (male and female coloration) play important parts in fish sexual behaviors and reproduction, further study could investigate the effects of transient exposure to DEHP during early life stages on the development of the neuro-endocrine system for sexual behaviors.

In this study, significant reductions in egg production and fertilization success were observed in adult fish transiently exposed to DEHP during early development (Fig. 4). Our results contradicted with those reported by Foran et al. [57] and Bhandari et al. [41], in which exposure of Japanese medaka to EE2 or bisphenol A (BPA) during embryonic development resulted in no change in fertilization success of the F_0_ adults. Foran et al. [57] exposed hatched larvae to EE2 at concentrations ranged from 0.0002 to 2 ppb for 2 weeks, while Bhandari et al. (2015) exposed 8 hpf eggs to 100 ppb BPA or 0.05 ppb EE2 for 7 days. Results of this study suggest that exposure to ultralow doses DEHP, which believes is 100 times less potent than EE2 [58], in early stages of development (from 4 hpf to 21 days old) is more harmful to adult reproduction success in F_0_ generation than by exposure to EE2 or young hatchlings alone. It is worth noting that in this study, although significant reductions in egg production and fertilization success were observed in adult fish transiently exposed to DEHP during early development, hatch rates (i.e., no. successful hatch out/no. successful fertilization) were not significantly affected (Fig. 4). Results of this study imply that although transient exposure to DEHP during early development irreversibly inhibited spermatogenesis and oogenesis in the adult fish (F_0_), development of embryos (F_1_ generation) did not appear to be affected, as almost all fertilized eggs managed to hatch out successfully (Fig. 5). Although lately, transgenerational effects have been reported in zebrafish and Japanese medaka exposed to high levels of BPA [41,59], the transgenerational effects on transient exposure to environmentally realistic concentrations of DEHP and other endocrine disruptors on the F_1_, F_2_ and F_3_ generations remain virtually unknown. Recently, exposure to DEHP has been found to significantly alter the expression of certain genes in the male gonads via DNA methylation on their promoter regions, thereby reducing sperm quantity and quality [48,60]. Further studies should be conducted to investigate if transient exposure to ultralow doses of DEHP during early development also results in similar DNA methylation statuses and whether these alterations are reversible or not in later generations as to provide a better understanding on the mode of actions of DEHP on fish development and reproduction.

## 5 Conclusion

To better protect the environment and human health, the Canadian Council of Ministers of the Environment recommends an upper limit of 16 ppb DEHP in surface water [61] and the World Health Organization recommends an upper limit of 8 ppb DEHP in drinking water [62]. Results of this study clearly demonstrated that transient exposure to 0.001 ppb DEHP (8000 times lower than the WHO guideline and 16,000 times lower than the CCMC guideline) during sensitive time windows of development irreversibly affected adult fish reproduction via upsetting oogenesis and spermatogenesis. Therefore, more studies are urgently needed to provide a better understanding on the effects of transient exposure to ultralow/environmentally realistic doses of DEHP on embryonic development and their subsequent effects on the adult reproduction, so as to adequately protect sensitive environmental species, our ecosystem and human health.

## Declaration of Competing Interest

The authors declare that they have no known competing financial interests or personal relationships that could have appeared to influence the work reported in this paper.

## Author contributions

B.B.H.Y., and A.B.Q designed the experiment. A.B.Q. performed the experiments. B.B.H.Y. and A.B.Q. analyzed dat. All authors prepared and reviewed the manuscript.

## CRediT authorship contribution statement

**Bonny Bun Ho Yuen:** Conceptualization, Writing - review & editing, Funding acquisition. **Anna Boya Qiu:** Visualization, Validation, Investigation, Methodology, Formal analysis. **Bruce Hao Chen:** .
